# Diabetes Medication Metformin Inhibits Osteoclast Formation and Activity in *In Vitro* Models for Periodontitis

**DOI:** 10.3389/fcell.2021.777450

**Published:** 2022-01-13

**Authors:** Lucy Y. Tao, Katarzyna B. Łagosz-Ćwik, Jolanda M.A. Hogervorst, Ton Schoenmaker, Aleksander M. Grabiec, Tim Forouzanfar, Fridus A. van der Weijden, Teun J. de Vries

**Affiliations:** ^1^ Department of Periodontology, Academic Centre for Dentistry Amsterdam, University of Amsterdam and Vrije University Amsterdam, Amsterdam, Netherlands; ^2^ Amsterdam University College, University of Amsterdam and Vrije University Amsterdam, Amsterdam, Netherlands; ^3^ Department of Microbiology, Faculty of Biochemistry, Biophysics and Biotechnology, Jagiellonian University, Kraków, Poland; ^4^ Department of Oral Cell Biology, Academic Centre for Dentistry Amsterdam, University of Amsterdam and Vrije University Amsterdam, Amsterdam, Netherlands; ^5^ Department of Oral and Maxillofacial Surgery and Oral Pathology, Amsterdam UMC, Amsterdam, Netherlands

**Keywords:** periodontitis, osteoclasts, diabetes, metformin, peridontal ligament

## Abstract

Diabetes and periodontitis are comorbidities and may share common pathways. Several reports indicate that diabetes medication metformin may be beneficial for the periodontal status of periodontitis patients. Further research using appropriate cell systems of the periodontium, the tissue that surrounds teeth may reveal the possible mechanism. Periodontal ligament fibroblasts anchor teeth in bone and play a role in the onset of both alveolar bone formation and degradation, the latter by inducing osteoclast formation from adherent precursor cells. Therefore, a cell model including this type of cells is ideal to study the influence of metformin on both processes. We hypothesize that metformin will enhance bone formation, as described for osteoblasts, whereas the effects of metformin on osteoclast formation is yet undetermined. Periodontal ligament fibroblasts were cultured in the presence of osteogenic medium and 0.2 or 1 mM metformin. The influence of metformin on osteoclast formation was first studied in PDLF cultures supplemented with peripheral blood leukocytes, containing osteoclast precursors. Finally, the effect of metformin on osteoclast precursors was studied in cultures of CD14^+^ monocytes that were stimulated with M-CSF and receptor activator of Nf-κB ligand (RANKL). No effects of metformin were observed on osteogenesis: not on alkaline phosphatase activity, Alizarin red deposition, nor on the expression of osteogenic markers RUNX-2, Collagen I and Osteonectin. Metformin inhibited osteoclast formation and accordingly downregulated the genes involved in osteoclastogenesis: RANKL, macrophage colony stimulating factor (M-CSF) and osteoclast fusion gene DC-STAMP. Osteoclast formation on both plastic and bone as well as bone resorption was inhibited by metformin in M-CSF and RANKL stimulated monocyte cultures, probably by reduction of RANK expression. The present study unraveling the positive effect of metformin in periodontitis patients at the cellular level, indicates that metformin inhibits osteoclast formation and activity, both when orchestrated by periodontal ligament fibroblasts and in cytokine driven osteoclast formation assays. The results indicate that metformin could have a systemic beneficiary effect on bone by inhibiting osteoclast formation and activity.

## Introduction

There are indications that diabetes patients have an increased risk of developing periodontitis ((Nascimento et al., 2018; Verhulst et al., 2019). Therefore, common pathogenesis pathways may exist. Periodontitis is the inflammation-related disease. It is characterized by degradation of alveolar bone that surrounds teeth, a process activated by recruited multinucleated osteoclasts. From a pharmacological point of view one could argue that medication that is used for diabetes may also benefit periodontitis patients. Metformin could be such a pleiotropic drug, showing positive effects not only in diabetes, but also in osteoporosis and periodontitis (Ursini et al., 2018). Its principal mode of action is as insulin sensitizer. It lowers the blood sugar level, which makes it a suitable drug for treating diabetes type II patients. It is a biguanid that has been used as an insulin sensitizer for more than 50 years (Halimi, 2006). The last 10 years have shown that metformin could be beneficial in various bone diseases as well, including periodontitis (Chandran, 2017).

Diabetes adversely affects a variety of organ systems in the body, including bones. It diminishes bone-mineral density and therefore weakens bone strength (Bak et al., 2010). When blood glucose levels increase, cartilage quality and quantity decrease (Mannucci and Dicembrini 2015). Diabetes patients have lower bone quality, and metformin may reverse these effects on bone, as shown in preclinical animal studies and clinical applications (Bahrambeigi 2019). It is therefore hypothesized that insulin stimulates osteoblast differentiation and bone matrix synthesis.

A beneficiary effect of metformin on bone has also been studied in periodontitis models (Bak et al., 2010; Araújo et al., 2017; Sun et al., 2017). Bak et al. investigated the effects of metformin on the progression of periodontitis in rats with ligature-induced periodontitis. Metformin reduced inflammatory cell infiltration and alveolar bone loss in periodontal tissues (Bak et al., 2010). This and other findings (Araújo et al., 2017) suggest that low doses of metformin reduce bone loss. Sun et al. found a higher number of osteoclasts in diabetic rats compared to non-diabetic rats (Sun et al., 2017). In the same study, it was shown that metformin decreased the number of osteoclasts compared to untreated diabetic rats (Sun et al., 2017). Gingival fibroblasts, when exposed to lipopolysaccharide (LPS) from periodontopathic bacterium *Porphyromonas gingivalis*, respond to metformin by severely lowering the expression of inflammatory cytokines IL-6, IL-1β and TNF-α, demonstrating an anti-inflammatory function of metformin (Kang et al., 2017). Calcium phosphate scaffolds with incorporated metformin showed increased osteogenic activity by periodontal ligament fibroblasts, which holds promise for use of metformin in bone regeneration (Zhao et al., 2019). These mouse studies urge extrapolating research using human cell systems on both the bone formation and bone degradation site, hereby elucidating the beneficiary effect of metformin in the human context.

Clinically, metformin has been applied in experimental treatments of periodontitis patients. Metformin was applied in gels at the site of alveolar bone loss. Patients were treated with a gel consisting of 0.5, 1 or 1.5% metformin locally at the site of bone loss. 10 μL of gel was injected into the periodontal pockets, and during the trial time of 6 months, gingival crevicular fluid was collected for analysis (Pradeep et al., 2013). Comparing all metformin test groups to the control, periodontal parameters significantly improved (Pradeep et al., 2013). Rao et al. treated smokers with chronic periodontitis using 1% metformin gel in a clinical trial (Rao et al., 2013) and showed similar improvements. Subsequent follow-up studies with slight modifications resulted in similar results (Pradeep et al., 2015; Pradeep et al., 2016).

These promising results with metformin in animal studies as well as in clinical studies urge mechanistic insight using a relevant cell system. Periodontal ligament fibroblasts anchor teeth in bone and also play a crucial role in both osteogenesis and osteoclastogenesis, processes that can be elegantly mimicked *in vitro* (De Vries et al., 2017; Ruppeka-Rupeika et al., 2018; De Vries et al., 2019; Karlis et al., 2020; Loo-Kirana R.; Gilijamse M.; Hogervorst J., 2021). Support for the relevance of periodontal ligament fibroblasts comes from an animal study where periodontal ligament fibroblasts were shown to play a key role in alveolar bone regeneration (Ren et al., 2015).

In the present study, we assessed whether metformin has an alleged positive effect on osteogenesis by culturing periodontal ligament fibroblasts in the presence of osteogenic medium and metformin. The effect of metformin on mineralization was assessed by quantifying alkaline phosphatase activity, by staining deposited mineral with Alizarin red staining and by gene expression of osteogenic transcription factor RUNX-2, matrix protein collagen type I and bone matrix protein Osteonectin. The effect of metformin on osteoclastogenesis was assessed in co-cultures of periodontal ligament fibroblasts and peripheral blood mononuclear cells (PBMCs) containing osteoclast precursors. Here, the formation of multinucleated cells was assessed after staining osteoclast characteristic tartrate resistant acid phosphatase (TRAcP) and by counting the nuclei. Since osteoclast-like cells that are formed in these cocultures do not resorb bone (De Vries et al., 2006; Sokos et al., 2015), we further assessed the effect of metformin on osteoclast activity by culturing CD14^+^ monocytes in the presence of M-CSF and RANKL, a cocktail yielding osteoclasts.

## Materials and Methods

### Cell Cultures

The periodontal ligament fibroblasts were isolated from nine different donors who had their wisdom teeth extracted for medical reasons. Sampling from the donors was conducted at Amsterdam University Hospital, location VU University Hospital under supervision of TF. The studies involving human participants were reviewed and approved by the Medische Etische Toetsingscommissie VU Medisch Centrum, Amsterdam, the Netherlands. The teeth containing periodontal ligament fibroblasts were surgical waste control material for osteogenic differentiation of adipose tissue derived stem cells.

All the individuals were informed that the cells retrieved from the surgical waste material was used for research on bone formation and bone degradation. A signed informed consent and samples were coded to guarantee the anonymity of the donors as required by Dutch law. The use of buffy coats from the blood bank (Sanquin, Amsterdam) was under ethical committee number NVT230.01. Researchers handling the fibroblasts (LT, KL-C, TJdV) could not in any way trace back the identity of the donors. Cells were scraped off the middle one third of the root and were propagated until passage three and subsequently frozen in liquid nitrogen. Cells from passage five were used in all experiments.

### Osteogenesis and Osteoclastogenesis Assays Using Periodontal Ligament Fibroblasts

Confluent flasks at passage four were trypsinized and cells were seeded at day -1 in 48 wells plates at 1.5∙10^4^ cells per well for osteoclastogenesis assays or 3∙10^4^ cells per well for osteogenesis assays. At day 0, the start of the experiment, the culture medium was removed from the 48-well plates. For the osteogenesis-assay plates, 0.4 ml mineralisation medium - composed of DMEM, 10% FCI, 1% PSF, 50 μg/ml ascorbic acid (Sigma), 10 nM β-Glycerophosphate (Sigma), and different metformin concentrations (or the solvent volume of sterile water, in the case of 0 mM metformin) were added to the wells. For osteoclastogenesis assays, peripheral blood mononuclear cells (PBMCs) derived from a buffy coat (Sanquin, Amsterdam) were added as source containing osteoclast precursors. ACTA has an agreement with bloodbank Sanquin to use buffy coats for research purposes. Metformin (Sigma Aldrich, D150959) was dissolved in demineralized water and added to cultures in a range between 0 and 1.0 mM, according to concentrations used in the literature.

For the osteoclastogenesis-assay plates, 0.2 ml containing 5∙10^5^ PBMCs in culture medium were added to the wells as well as 0.2 ml of metformin-consisting medium. During the 21 days duration of the experiment, culture- and mineralization-medium with the appropriate concentration of metformin was replenished twice a week.

### DNA Concentration and Alkaline Phosphatase Activity

Cells were harvested at day 0 and day 14 of culturing. In order not to sample dead cells, the adherent cells were washed twice with PBS. Next, they were lysed in 150 µl MilliQ per well and stored in −20°C. Prior to analysis, the plates underwent three cycles of freeze-thawing. Alkaline phosphatase (ALP) activity was measured using 4-nitrophenyl phosphate disodium salt (Merck, Darmstadt, Germany) at pH 10.3 as a substrate for ALP according to the method described by Bastidas-Coral (Bastidas-Coral et al., 2016). After incubation of 60 min at 37°C, the reaction was stopped with sodium hydroxide. Absorbance was measured with 405 nm with a Synergy HT spectrophotometer (BioTek Instruments Inc., Winooski, VT). DNA concentration (ng/ml) was measured using CyQuant Cell Proliferation Assay Kit (Molecular Probes, Leiden, Netherlands) mixed with lysis buffer. Fluorescence was measured at 485 nm excitation and 528 nm emission with a Synergy HT spectrophotometer (BioTek Instruments Inc., Winooski, VT, United States). ALP activity was expressed as ALP per DNA (nMol/ng).

### Alizarin Red Staining

Alizarin red staining was performed after 14 or 21 days to analyze the mineral deposition. 2% Alizarin red S at pH 4.3 (Sigma-Aldrich) was used for staining. Cells were fixed for 10 min in 4% formaldehyde and rinsed with deionized water before adding 300 µl of 2% Alizarin solution per well. After incubation of 15 min at room temperature, the cells were washed with deionized water and air-dried. Mineral deposition was visualized as red nodules.

### Quantitative Polymerase Chain Reaction

Quantitative polymerase chain reaction (qPCR) analysis was performed for osteogenesis and osteoclastogenesis at day 0 and 14 or 21. At these time points the culture medium was removed and 200 µl RNA lysis buffer (Qiagen, Hilden, Germany) was added per well. Subsequently, the 48-well plates were stored at −80°C until further use. RNA isolation was performed with Qiagen RNeasy Mini kit according to the manufacturer’s instructions. The RNA concentration and quality was determined using absorption read at 260 and 280 nm with Synergy HT spectrophotometer (BioTek Instruments Inc., Winooski, VT). RNA was reverse transcribed to cDNA with the MBI Fermentas cDNA synthesis Kit (Vilnius, Lithuania). Oligo(dT)18 and D(N)6 were used as primers. Real time primers were designed for several genes. PCR was performed on the LC480 light cycler (Roche, Basel, Switzerland). Beta-2-microglobulin (β2M) was used as a housekeeping gene for the osteogenesis markers, while hypoxanthine phosphoribosyltransferase 1 (HPRT1) was used as housekeeping gene for the osteoclastogenesis and osteoclast makers. Gene expression was normalized for β2M or HPRT1 expression following the comparative threshold (Ct) method. ∆C (Ct _gene of interest_ – Ct _housekeeping gene_) was calculated and relative expression of the genes was determined as 2^−∆Ct^. The primer sequences are listed in Table 1.

**TABLE 1:** primers used.

Genes	Primer sequence 5′-3′	Amplicon length (bp)	ENSEMBL gene ID
Housekeeping gene			
HPRT	Fw TgA​CCT​TgA​TTT​ATT​TgC​ATA​CC	101	ENSG00000165704
	Rv CGA​gCA​AgA​CgT​TCA​gTC​CT		
Β2M	Fw CgACggCCgCTgTCA	100	ENSG00000160255
	Rv TgT​TgT​TTT​CAg​CCA​gCT​TgT​g		
Osteogenesis			
RUNX2	Fw CCA​gAA​ggC​ACA​gAC​AgA​AgC​T	156	ENSG00000124813
	Rv Agg​AAT​gCg​CCC​TAA​ATC​ACT		
Collagen I	Fw TCC​AAC​gAg​ATC​gAg​ATC​C	190	ENSG00000108821
	Rv AAgCCgAATTCCTggTCT		
Osteonectin	Fw TAC​ATC​ggg​CCT​TgC​AAA​TAC	100	ENSG00000231061
	Rv Agg​gTg​ACC​Agg​ACg​TTC​TTg		
Osteoclastogenesis		67	ENSG00000141655
RANK	Fw CCT​ggA​CCA​ACT​gTA​CCT​TCC​T		
	Rv ACC​gCA​TCg​gAT​TTC​TCT​gT	60	ENSG00000120659
RANKL	Fw CAT​CCC​ATC​Tgg​TTC​CCA​TAA		
	Rv gCCCAACCCCgATCATg	100	ENSG00000164761
OPG	Fw CTgCgCgCTCgTgTTTC		
	Rv ACA​gCT​gAT​gAg​Agg​TTT​CTT​CgT	100	ENSG00000184371
M-CSF	Fw CCg​Agg​Agg​TgT​Cgg​AgT​AC		
	Rv AAT​TTg​gCA​CgA​ggT​CTC​CAT		
DC-STAMP	Fw ATT​TTC​TCA​gTg​AgC​AAg​CAg​TTT​C	101	ENSG00000164935
	Rv gAA​TCA​Tgg​ATA​ATA​TCT​TgA​gTT​CCT​T		
TRAcP	Fw CAC​AAT​CTg​CAg​TAC​CTg​CAA​ggA​T	128	ENSG00000102575
	Rv CCC​ATA​GTg​gAA​gCg​CAg​ATA		
CD14	Fw ggT​TCg​gAA​gAC​TTA​TCg​ACC​AT	109	ENSG00000170458
	Rv TCA​TCg​TCC​AgC​TCA​CAA​ggT		
Cathepsin K	Fw CCA​TAT​gTg​ggA​CAg​gAA​gAg​AgT​T	149	ENSG00000143387
	Rv TgC​ATC​AAT​ggC​CAC​AgA​gA		
Nfatc1	Fw AgC​AgA​gCA​Cgg​ACA​gCT​ATC	143	ENSG00000131196
	Rv ggT​CAg​TTT​TCg​CTT​CCA​TCT​C		
Cox1	5′ AgT​ACA​gCT​ACg​AgC​AgT​T 3′	100	ENSG00000229899
	5′ gAg​ACT​CCC​TgA​TgA​CAT​CC 3′		
Cox2	5′ gCA​TTC​TTT​gCC​CAg​CAC​TT 3′	100	ENSG00000267084
	5′ AgA​CCA​ggC​ACC​AgA​CCA​AAg​A 3′		

### TRAcP Staining and Osteoclast Counts

Plates for detecting tartrate resistant acid phosphatase (TRAcP) activity staining were harvested in duplicate on t = 21. Cells were washed with PBS and fixed with 4% PBS buffered formaldehyde for 10 min before being stored with PBS at 4°C. After washing the cells with water at 37°C, TRAcP staining solution was made consisting of Fast Garnet GBC base, sodium nitrate, acetate, naphthol AS-BI and tartrate in AD on 37°C, using the leukocyte acid phosphate kit (Sigma) following the manufacturer’s instructions. The cells were counterstained with DAPI (diamidino-2phenylindole dihydrochloride). A distinction was made between cells with 3-5 nuclei, and cells with ≤6 nuclei. For analyses, the average of the number counted in the duplicate wells per patient was used.

### Blood Cell Isolation

Buffy coats (Sanquin, Amsterdam, Netherlands) of healthy donors were diluted 1:1 in 1% PBS-citrate (pH 7.4). Thereafter, 25 ml of diluted blood was carefully layered on 15 ml Lymphoprep (Axisshield Po CAS, Oslo, Norway) and centrifuged for 30 min at 800 x G without brake. The interphase containing the PBMCs was collected and washed three times in 1% PBS-citrate by spinning down the cells at 400 g with full brakes and by decanting the supernatant. Finally, the cells were recovered in culture medium.

### Monocyte Isolation

CD14^+^ monocytes retrieved from peripheral blood were used in experiments where osteoclasts were grown using M-CSF and RANKL instead of fibroblasts and PBMCs. Here, CD14^+^ cells were isolated using CD14^+^ microbeads (Miltenyi, Bergisch Gladbach, Germany) according to a previously described method (Ten Harkel et al., 2015). CD14^+^ monocytes were cultured for 3 days in M-CSF (25 ng/ml), followed by 10 ng/mL M-CSF and 10 ng/ml RANKL until 21 days.

### Bone Resorption

Bone resorption was analyzed in cultures on bone after a culture period of 21 days. After this period, the cells present on the bovine cortical bone slices were removed with 0.25 M NH_4_OH followed by sonication in ice water for 30 min. The slices were washed in distilled water, incubated in a saturated alum solution, washed in distilled water, and stained with Coomassie Brilliant blue. The surface areas of individual resorption pits were measured using Image-Pro Plus software (Media Cybernetics, Silver Spring, MD).

### Statistical Analysis

The effects on the cells between different metformin concentrations over time were compared using one way ANOVA followed by the non-parametric Friedman test, using GraphPad Prism software. Dunn’s multiple comparison test was done as a post-test. A *p* value of *p ≤ 0.05 was chosen to determine the level of significance.

## Results

### Metformin Titration in Periodontal Ligament Mediated Osteogenesis

In order to establish whether metformin plays a role in periodontal ligament driven osteogenesis, we added 5-fold dilutions (0.008–1 mM) of metformin in mineralization assays (n = 3 donors). As first measurement, we assessed whether metformin could influence proliferation, measure of how metformin could influence metabolic activity such as proliferation. Metformin had no effect on proliferation (Supplementary Figure S1A), nor on the alkaline phosphatase activity per cell (Supplementary Figure S1B). Alizarin red staining showed donor variability at 14 days, which vanished at 21 days (Supplementary Figure S1C). At higher magnification, the alizarin red staining appeared in nodule-like structures (Supplementary Figure S1D). No clear effect of metformin on mineralization was observed.

### Metformin Does Not Affect Periodontal Ligament Mediated Osteogenesis

**FIGURE 1:**
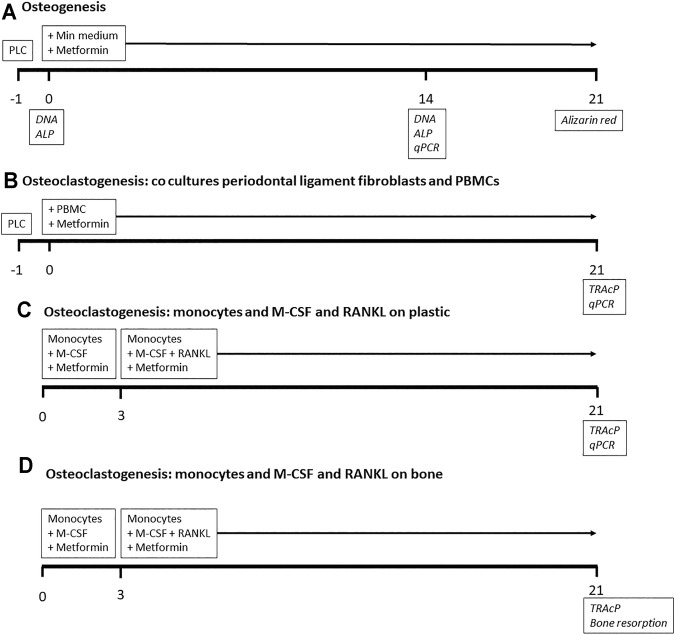
Outline of experiments. Timeline in days. For osteogenesis and osteoclastogenesis co culture experiments, periodontal ligament cells (PLC) were cultured 1 day before the start of the experiment. Metformin was added throughout all experiments. Analyses in italics at the days of sampling. PBMCs: peripheral blood mononuclear cells.

**FIGURE 2:**
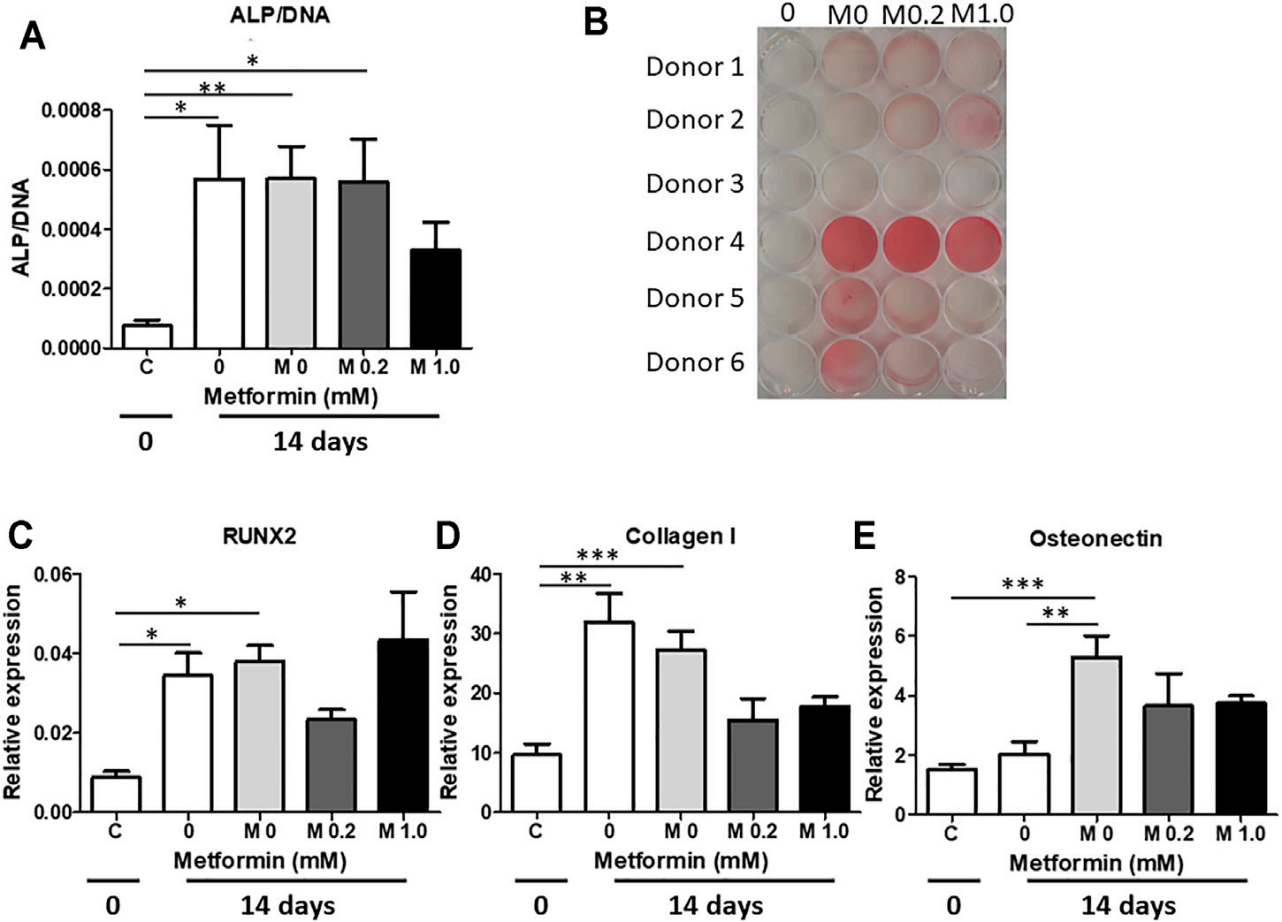
Metformin does not increase mineralization by periodontal ligament fibroblast cultures. Metformin was used in two concentrations (0.2 and 1.0 mM) in mineralization assays. **(A)** Alkaline phosphatase enzyme activity per cell at 0 **(C)** and 14 days (0 – normal medium without metformin; M0, M0.2 and M1.0 0, 0.2 and 1.0 mM Metformin in Mineralization medium). **(B)** Alizarin red staining at 21 days, **(C–E)**: genes expression of RUNX2 **(C)**, Collagen I **(D)** and Osteocalcin **(E)** at 0 days **(C)** and at 14 days (0 – normal medium without Metformin; M0, M0.2 and M1.0 0, 0.2 and 1.0 mM Metformin in Mineralization medium). *: *p* < 0.05; **: *p* < 0.01; ***: *p* < 0.001. All data are from n = 6 donors.

The above titration experiment showed that metformin was tolerated at high concentrations and seemed not to affect osteogenesis. All subsequent osteogenesis and osteoclastogenesis experiments were performed using a higher number periodontal ligament donors (n = 6) with 0.2 and 1.0 mM metformin. Compared to day 0, ALP enzyme was significantly higher at day 14, with no differences between normal medium, mineralization medium without or with metformin (Figure 2A). Alizarin red staining demonstrated greater variability compared to the donors of Figure 1, but again with no clear metformin effect (Figure 2B). qPCRs for early osteogenic marker RUNX 2 (Figure 2C), Collagen type I (Figure 2D) and late osteogenic marker Osteonectin (Figure 2E) showed an increase in expression over time (t = 0 vs. t = 14) for all genes. Only osteonectin expression differed between normal medium and mineralization medium at t = 14 days. Metformin had no effect on osteogenic gene expression. These experiments together showed that metformin did not affect osteogenic differentiation of periodontal ligament fibroblasts.

### Effect of Metformin in Periodontal Ligament- PBMC Co Cultures

**FIGURE 3:**
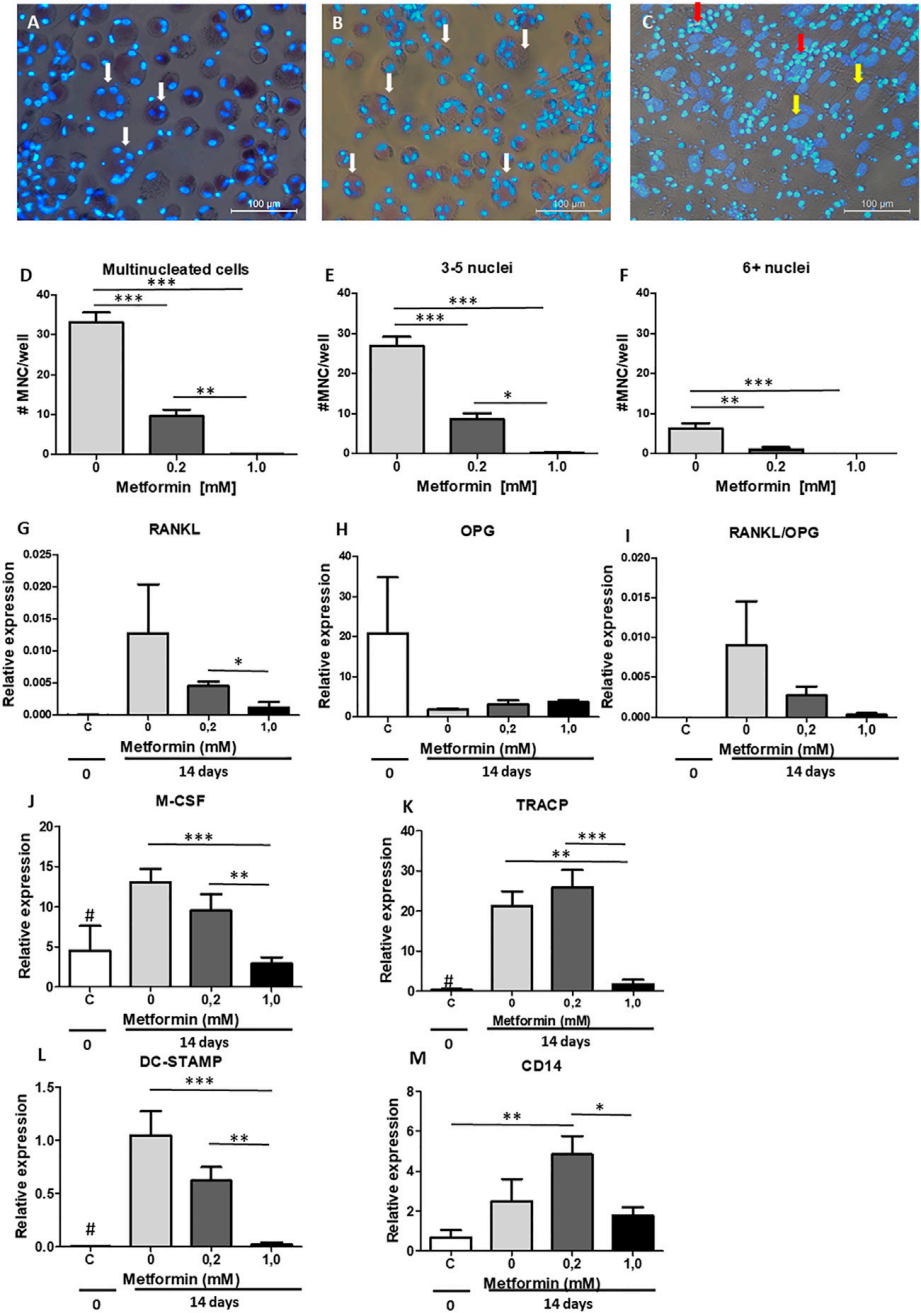
Metformin inhibits periodontal ligament fibroblasts mediated osteoclast formation. PBMC were cultured on periodontal ligament fibroblasts and osteoclast formation was assessed at 21 days after TRAcP staining **(A–C)**. **(A)** Control, without metformin, **(B)** 0.2 mM metformin and **(C)** 1.0 mM metformin. **(D–F)**: Inhibition of numbers of multinucleated cells (MNC) formed, both when assessing all multinucleated cells **(D**, >2 nuclei**)**, small multinucleated cells with 3-5 nuclei **(E)**, or larger multinucleated cells **(F**, 6+ nuclei**)**. **(G–M)**: Gene expression at 0 days **(C**, periodontal ligament fibroblasts without PBMCs**)** or 21 days (co-cultures periodontal ligament fibroblasts 0, 0.2 and 1.0 mM metformin) of RANKL **(G)**, OPG **(H)**, the ratio RANKL/OPG **(I)**, M-CSF **(J)**, TRACP **(K)**, DC-STAMP **(L)**, and CD14 **(M)** is inhibited by metformin. All results are from n = 6 periodontal ligament fibroblast donors. White arrow in **A** and **B**: multinucleated cells, Yellow arrow in **C**: fibroblast nucleus, Red arrow in **C**: PBMCs that are not fused and TRACP negative. # in **G–I**: Control significantly lower (*p* < 0.05) expression than 0 and 0.2 mM metformin in co-cultures. *: *p* < 0.05; **: *p* < 0.01; ***: *p* < 0.001. All data are from n = 6 donors. Osteoclast assay **(A–F)** was performed twice, obtaining similar results, with two groups of six different donors (by LT and KL), results from one experiment are shown.

As previously shown (De Vries et al., 2017; Ruppeka-Rupeika et al., 2018; Loo-Kirana R.; Gilijamse M.; Hogervorst J., 2021), the periodontal ligament plays a role in both osteogenesis and osteoclastogenesis. We next assessed whether metformin could inhibit osteoclast formation by making use of periodontal ligament fibroblasts-PBMC co-cultures. To determine the number of osteoclasts, cells were stained with TRAcPand counterstained with DAPI to visualize nuclei. Cells containing ≥3 nuclei are considered as osteoclasts. Metformin was used in concentrations that did not affect osteogenesis: 0.2 and 1.0 mM. A decrease in osteoclast formation was observed with both concentrations (Figures 3A–C), affecting total number of cells containing more than two nuclei (Figure 3D), the smaller ones containing 3–5 nuclei (Figure 3E) as well as the larger ones with six and more nuclei (Figure 3F). Expression of genes associated with osteoclast formation such as RANKL (Figure 3G), OPG (Figure 3H), M-CSF (Figure 3J), and osteoclast genes TRAcP (Figure 3K) and DC-STAMP (Figure 3L) displayed a similar pattern of decreased expression in the presence of 0.2 or 1.0 mM metformin. RANKL was not detetable by qPCR in monocyte cultures stimulated with M-CSF and RANKL. OPG was detected with high Ct values, with an unreliable relative expression between 0.0002 and 0.001. These results confirm what we have previously seen (De Vries et al., 2006; Bloemen et al., 2010), showing that OPG and RANKL were solely expressed by periodontal ligament fibroblasts in co culture. Monocyte marker CD14 was elevated at 0.2 mM of metformin (Figure 3M), suggesting that the inhibitory effect of metformin was due to a decrease in osteoclast differentiation rather than loss of precursor cells.

### Metformin Inhibits M-CSF and RANKL Mediated Osteoclast Formation and Bone Resorption

Although periodontal ligament fibroblasts contribute to osteoclast formation, M-CSF and RANKL are required for the generation of bone resorbing osteoclasts (De Vries et al., 2006). In order to study the effect of metformin on M-CSF and RANKL mediated osteoclast formation and bone resorption, CD14^+^ cells were seeded on plastic or bone for assessing the impact of metformin on osteoclastogenesis. In a separate seeding on bone, the effect on bone resorption was assessed.

**FIGURE 4:**
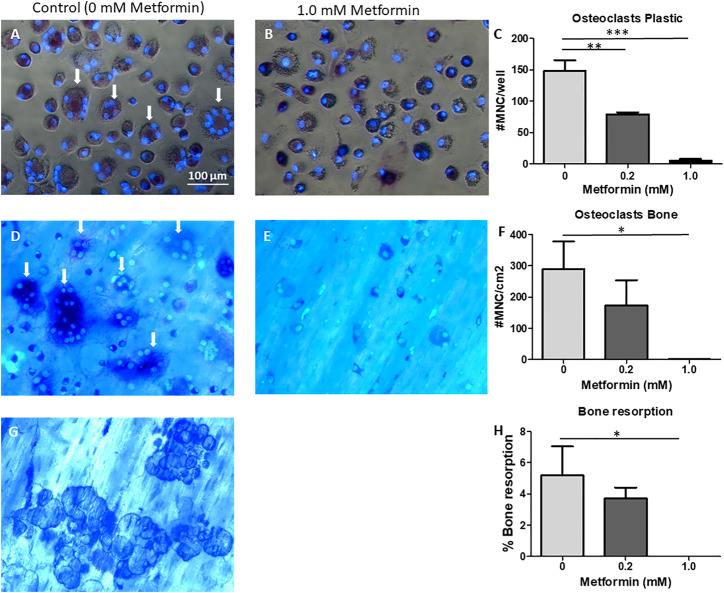
Metformin inhibits M-CSF and RANKL driven osteoclast formation. CD14^+^ cells as source of osteoclast precursors were seeded on plastic **(A–C)**, or on bone **(D–H)** and cultured with M-CSF and RANKL, without or with 0.2 or 1.0 mM Metformin. Micrographs of cells on plastic **(A,B)** or on cortical bone slices **(D,E)** or bone resorption pits **(G)**. Quantification of cell counts on plastic **(C)**, bone **(F)**. **(H)**: Quantification of bone resorption. Quantitative data were from quadruplicate seeding. *: *p* < 0.05; **: *p* < 0.01; ***: *p* < 0.001. Scale bar in **A** applies to all micrographs. Arrows point to TRAcP multinucleated cells/osteoclasts.

As in the co-cultures, metformin inhibited osteoclast formation in a dose dependent manner on plastic (Figures 4A–C) as well as on bone (Figures 4D–F). Note that monocytes remained mononuclear at 1.0 mM metformin (Figures 4B,E). In parallel to decreased osteoclast formation on bone slices, bone resorption was abolished at 1.0 mM metformin (Figure 4H).

**FIGURE 5:**
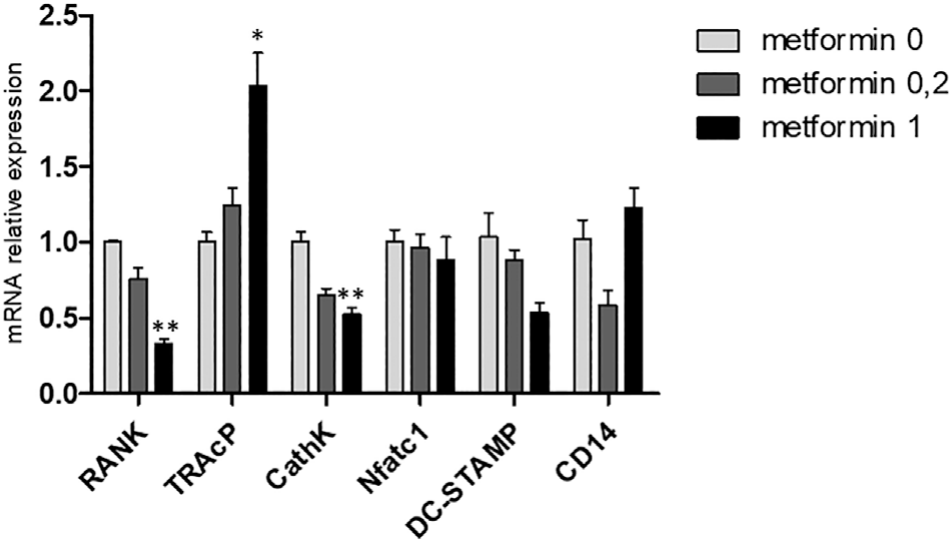
Metformin affects gene expression in M-CSF and RANKL driven osteoclast cultures. Gene expression of RANK, TRAcP, Cathepsin K, NFATc1, DC-STAMP and CD14 at 21 days. Expression of the control, metformin 0, was set at 1.0. Decreased expression of RANK and Cathepsin K was observed and increased expression of TRAcP. *:*p* < 0.05, **:*p* < 0.01.

Gene expression of osteoclast genes RANK and CathK was inhibited at high dose of metformin (Figure 5). Peculiarly, the reverse was true for TRAcP expression (Figure 5), where higher expression was observed when cultured with 1.0 mM metformin. Expression of DC-STAMP, Nfatc1 and CD14 was not altered by metformin.

Gene expression of mitochondrial markers COX1 and COX2 tended to be lower at 1.0 mM metformin (Supplementary Figure S2 (non-significant).

## Discussion

Various animal (Bak et al., 2010; Araújo et al., 2017) and clinical studies (Pradeep et al., 2013; Pradeep et al., 2015; Pradeep et al., 2016; Pradeep et al., 2017; Kurian et al., 2018) have pointed out a beneficiary effect of metformin in down modulating the severity of periodontitis, when induced or when used as a clinical adjuvant improving bone health and periodontal status. The present study contributes to an understanding at the cellular level using the appropriate model of periodontal ligament fibroblasts. Using two confirmatory assays, results unequivocally point out a role for metformin by inhibiting osteoclast formation and activity.

Our original hypothesis that metformin would have a positive effect on osteogenesis, has to be partially rejected and modified. Based on our results, metformin treatment did not induce alkaline phosphatase activity nor did it increase Alizarin Red staining. This is in line with a recent publication on the effect of metformin on periodontal ligament fibroblasts (Jia et al., 2020). In contrast to Jia et al., we did not observe a positive effect on osteogenic markers such as RUNX-2. Differences in periodontal ligament fibroblast heterogeneity, such as seen for Alizarin Red staining in Figure 2B could account for this difference, but also methodological differences, such as use of dexamethasone and a different concentration of ascorbic acid and glycerophosphate could give rise to such discrepancies. Concentrations that were used in our study did not affect proliferation, since no differences in DNA content were observed in the titration experiment (Suppl. Figure 1A). As in previous studies from our group (Karlis et al., 2020; Loo-Kirana et al., 2021), the observed Alizarin Red staining was due to so-called nodules or hot-spots of mineralization that formed on top of the fibroblasts (Figure 1D). Interestingly, mineralization medium only induced osteopontin expression when compared to normal medium, whereas RUNX-2, Collagen-I expression and the activity of Alkaline phosphatase was not influenced, not by mineralization medium nor by metformin. Clearly, most of the expression data revealed a time effect (t = 0 vs. 14 days), which possibly can be explained by a more confluent layer of cells, whichmay ignite these expressions. By nature, periodontal ligament fibroblasts express alkaline phosphatase abundantly (Groeneveld et al., 1995), which may explain why mineralization medium did not alter the expression.

Metformin inhibited osteoclast formation or osteoclastogenesis in the periodontal ligament-PBMC co cultures. Downregulation of typical osteoclastogenesis genes, such as RANKL and M-CSF, that are expressed by periodontal ligament fibroblasts as well as the downregulation of osteoclast gene DC-STAMP shows that metformin affects both the fibroblast side that orchestrate osteoclast formation (RANKL and M-CSF), as well as precursor cells (DC-STAMP). Downregulation of DC-STAMP most likely resulted in a lack of fusion (Figure 3C), where only mononuclear cells were seen in conjunction with fibroblasts. Concerning mechanistic insight, pleotropic effects of metformin beyond facilitating insulin’s glucose lowering effects should be considered (Ursini et al., 2018). It has been described in a cell culture using gingiva fibroblasts that metformin reduces the expression of inflammatory factors IL-1β, TNF-α and IL-6 (Wu et al., 2015; Kang et al., 2017). Recently, we showed that co-cultures of gingiva fibroblasts with leukocytes induces IL-1β and TNF-α (Moonen et al., 2018), but also periodontal ligament fibroblasts induce M-CSF, IL-1β and TNF-α early during osteoclast formation (Bloemen et al., 2010). These factors are adjuvant cytokines for osteoclast formation, since osteoclast formation can be inhibited by anti-TNF (De Vries et al., 2016; Zamri and De Vries, 2020) and stimulated by TNF-α (Cao et al., 2017) and IL-1β (Cao et al., 2016). *In vivo* evidence for metformin anti-inflammatory mode of action was obtained in a mouse model of rheumatoid arthritis (Fan et al., 2020) where it primarily affected IL-1β. Together with the downregulated crucial osteoclastogenesis factor RANKL (Kong et al., 1999) (Figure 3G), it seems likely that also other inflammatory osteoclastogenesis genes could be affected. Downregulation of RANKL was also seen in ovariectomized rats that were treated with metformin (Mai et al., 2011), who also showed that this brought down the number of osteoclasts *in vivo*. Co. cultures in the present study were performed with unsorted PBMCs, which makes it likely that also Th17 T-cells were present in the culture. These cells may contribute to osteoclast formation by interacting with osteoclast precursors (Moonen et al., 2018), producing the osteoclastogenesis activating cytokine IL-17 (Sprangers et al., 2016). Interestingly, it was shown that metformin lowers the Th17/Treg ratio (Son et al., 2014). Whether metformin affects this T-cell subset and thus contribute to the inhibitory effect remains to be investigated.

Metformin likewise inhibited monocytes in the absence of periodontal ligament fibroblasts to differentiate into osteoclasts, both on plastic and on bovine bone slices. Here, no resorptive activity was observed when the highest concentration of metformin was used. qPCR revealed cathepsin K and RANK to be downregulated by metformin, mRNAs that could not be identified as osteoclast precursor specific in the cocultures with periodontal ligament fibroblasts, since these molecules are also detectable in monocultures of periodontal ligament fibroblasts. These results indicate that metformin inhibits genes important for early differentiation events (RANK) as well as genes involved in differentiated osteoclasts, such as cathepsin K. One of the mechanistically interesting aspects that could explain the observed reduced osteoclast formation and function, is metformin’s mildly inhibitory effect on mitochondrial function (Apostolova et al., 2020). Recent studies interfering with osteoclast presence (Weivoda et al., 2020) could correlate osteoclast activity to a general energetic state. Abundance of mitochondria is a hallmark of osteoclasts, and interference with mitochondrial function interferes with osteoclast formation (Park-Min, 2019; Weivoda et al., 2020). Preliminary results indicate that 1.0 mM could have a downregulatory effect on the expression of mitochondrial markers COX1 and COX2 (Figure 5B). Contra intuitively, TRAcP was upregulated by metformin, which was also seen with 0.2 mM in the cocultures. This could indicate that metformin diversely affects the transcription of osteoclast genes: DC-STAMP and Cathepsin K were downregulated whereas TRAcP was upregulated, which is different from our previous work on mouse osteoclasts, where upregulation of all osteoclast genes was similar (De Vries et al., 2009). TRAcP is also expressed by the osteoclast’s mononuclear family members, macrophages and dendritic cells. Here, TRAcP may be of importance in bacterial clearance and general functionality of macrophages and dendritic cells (Haymon 2008). It is therefore feasible that metformin shifts the differentiation of monocytes towards macrophages and dendritic cells.

Concerning the balance of strong and weaker points of this study, we show with two assays that osteoclast formation and osteoclast activity are inhibited by metformin. 1) Metformin inhibited cell-cell mediated osteoclastogenesis, with the identification of various PDL related genes that are downregulated (M-CSF, RANKL). 2) In assays using only osteoclast CD14 precursors, it was shown that also in this “pure” system, osteoclast formation and activity is inhibited, concomitant with down regulation of RANK and Cathepsin K. A weak point in our study is that, although metformin did not interfere with cell numbers of periodontal ligament fibroblasts, it is not clear whether metformin may also induce secreted osteoclastogenesis inhibitory molecules. Also, the possible effects of metformin on the energy metabolism, such as in osteoclasts, needs further investigation.

**FIGURE 6:**
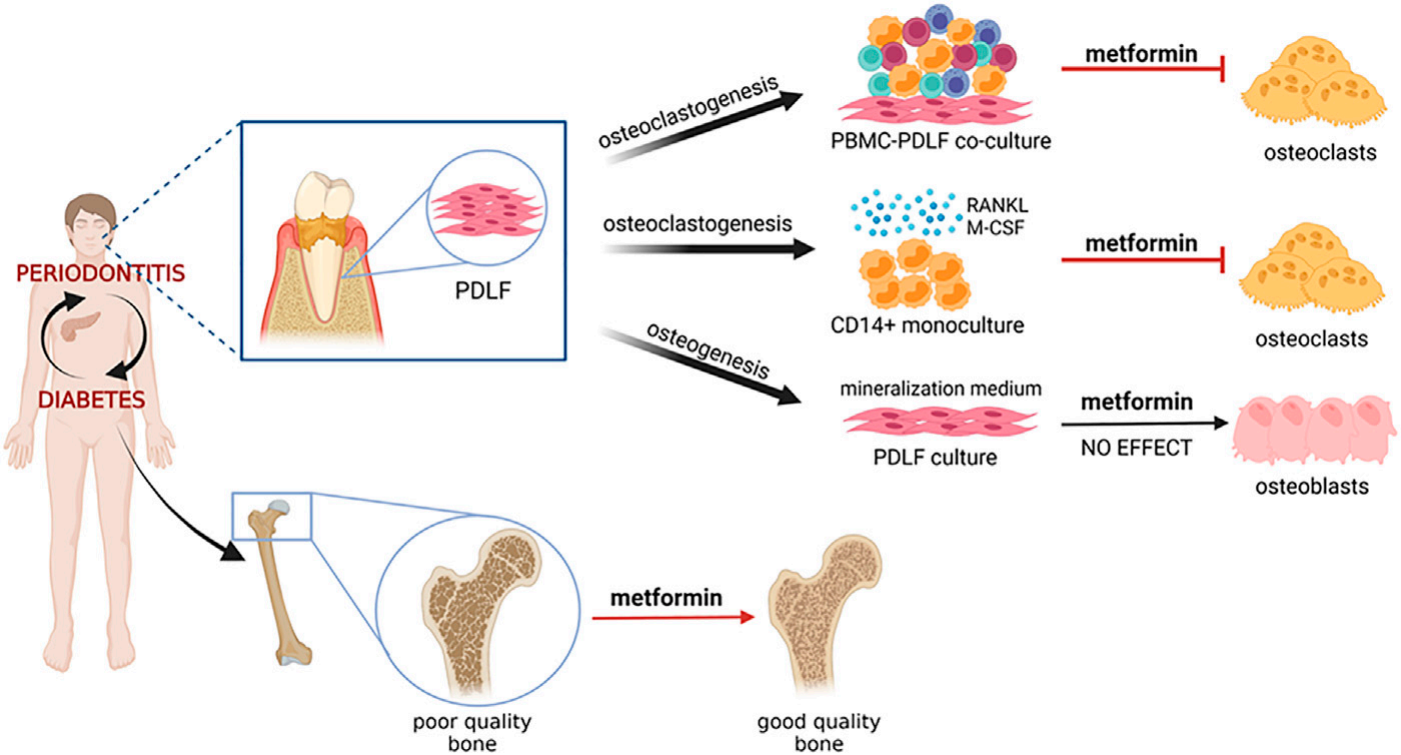
Graphical abstract. Diabetes and periodontitis are clinically associated.Mouse experimental periodontitis modelas and preliminary clinical studies show that diabetes drug metformin has beneficial effects on the periodontium, the tooth associated tissues including bone. This study has analyzed the effect of metformin on clinically relevant cell models: periodontal ligament derived fibroblasts (PDLF) that were either steered into osteogenesis pathways or into osteoclastogenesis pathways, together with peripheral blood monocnuclear cells (PBMCs) under continual exposure to metformin. The effect of metformin on osteoclast formation was further assessed with CD14^+^ monocytes that were subjected to osteoclast differentiation with osteoclastogenesis driving cytokines M-CSF and RANKL. Our results all indicate an inhibitory effect on osteoclast formation, which could, in a broader sense, be the mechanism that explains clinically observed bone improvement in metformin users.

Anti-aging has recently been added to the long list of metformin’s pleotropic effects (Hu et al., 2021; Mohammed et al., 2021). Increase in osteoclasts and osteoclastic activity can be seen as a result of aging, especially as a result of menopause. The present study therefore could add anti-osteoclast formation as one of the aspects of anti-aging. Metformin influence both the orchestrator of osteoclastogenesis, the periodontal ligament fibroblast, as well as the monocyte osteoclast precursor. Our results, summarized in Figure 6, illustrate that the alleged bone sparing mechanism of metformin could be due to inhibiting osteoclasts, rather than, or in parallel with increased osteogenesis.

## Data Availability

The raw data supporting the conclusions of this article will be made available by the authors, without undue reservation.

## Supplementary Material

Supplementary Figure S1 Titration of metformin in a mineralization assay. Periodontal ligament fibroblasts were subjected to increasing concentrations of metformin (five-fold dilutions from 1- 0.008 mM in mineralization medium. **A.** Effect of metformin on proliferation. DNA was measured at t=0, (C) and 0-1.0 in mineralization medium at t=14 days. #: t=0 Significantly different from all t=14 conditions, no differences between the metformin treated donor cells. **B.** Alkaline phosphatase per cell, metformin did not affect alkaline phosphatase activity per cell. **C.** Alizarin red staining was assessed for 3 donors after 14 and 21 days of culture. Heterogeneous staining between donors at 14 days, whereas all donors were positive at 21 days. No effects of metformin were observed. **D.** Micrograph of alizarin red staining, showing red, circular structures, reminiscent of nodules.

Supplementary Figure S2 Effect of metformin on COX1 and COX2 expression. Gene expression of mitochondrial enzymes COX1 and COX2, with tendency of downregulation (n.s.)
