# Supplementary figures and images for: Diabetes Medication Metformin Inhibits Osteoclast Formation and Activity in In Vitro Models for Periodontitis
Source: Front Cell Dev Biol. 2022 Jan 13;9:777450. doi: 10.3389/fcell.2021.777450 (PMC8793072; doi:10.3389/fcell.2021.777450)

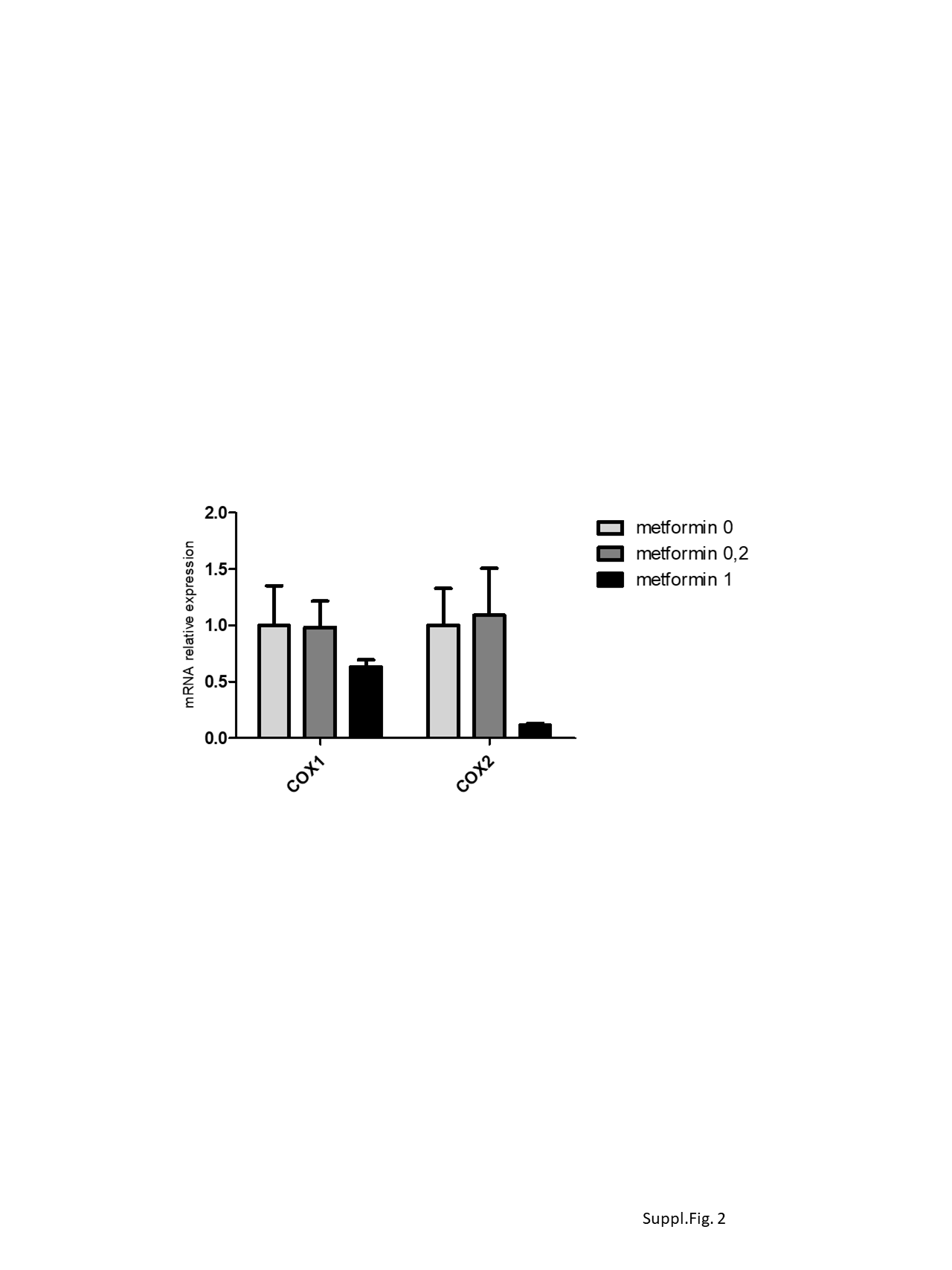

Supplement: Supplementary file 1 [file Image2.tif]

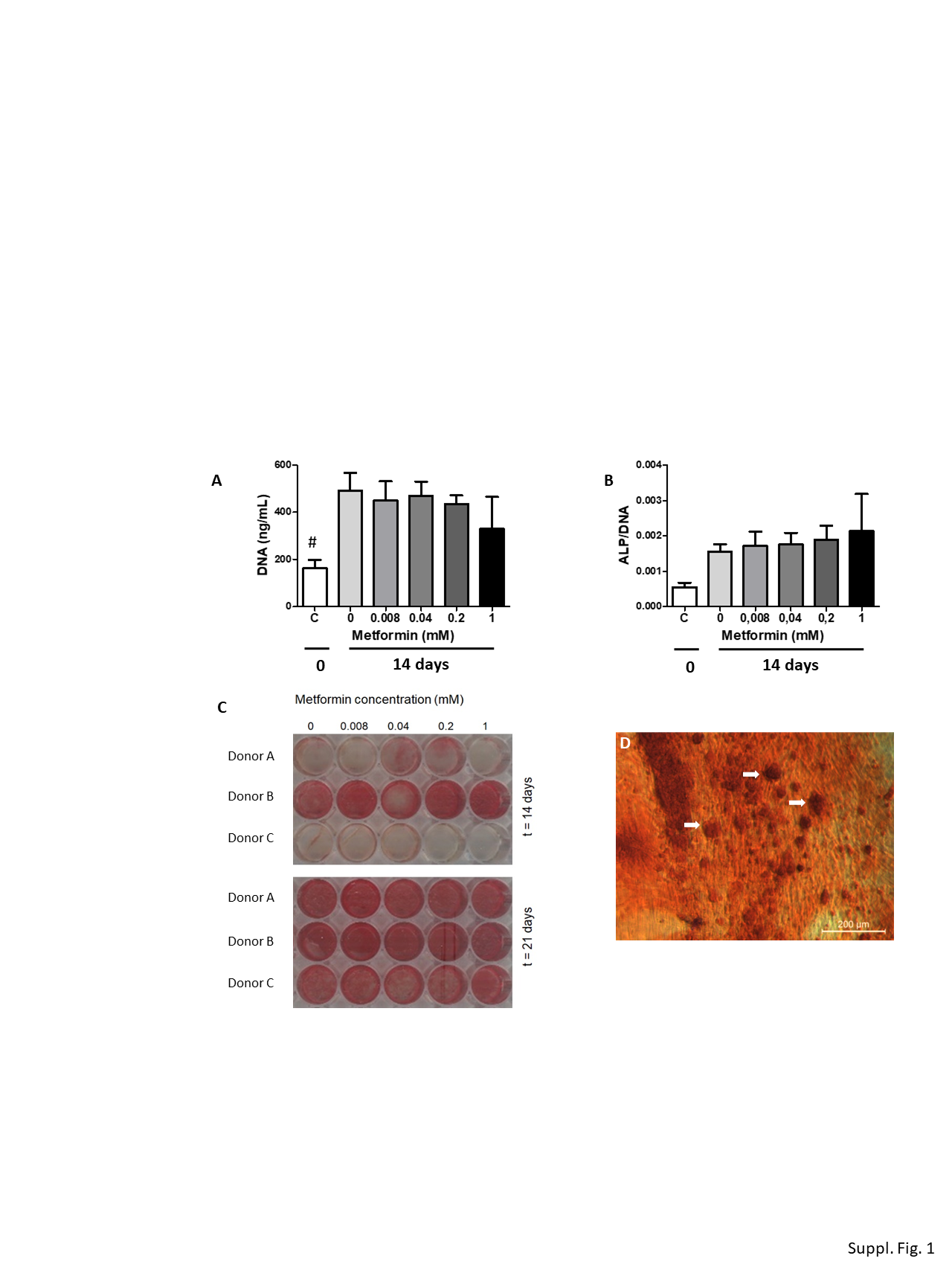

Supplement: Supplementary file 2 [file Image1.tif]
